# A landscape analysis of enablers and barriers to digital payments to health workers during large-scale immunisation campaigns in sub-Saharan Africa: a scoping review and in-depth interviews

**DOI:** 10.1080/16549716.2026.2675098

**Published:** 2026-05-21

**Authors:** Vincent Micheal Kiberu, Noel Namuhani, Elizabeth Ekirapa, Juliet Aweko, Charles Opio, Fredrick Kanobe, Peter Wakholi, Timothy Abuya, Maggie Mcconnell, Ahmed Hamani, Idil Hussein Jama, Michael Ediau, Segun Bello, David Adewol, Olufunmilayo I. Fawole, Petronille Acray-Zengbe, Daniel Arhinful, Georges Bediang, Adama Faye, Fatoumata Zahra Mohamed Mboup, Souleymane Ndiaye, Khadim Niang, Yves G. Obotela N’Sarhaza, Peter Waiswa

**Affiliations:** a Department of Epidemiology and Biostatistics, Makerere University School of Public Health, Kampala, Uganda; b Department of Health Policy Planning and Management, Makerere University School of Public Health, Kampala, Uganda; c Department of Computer Science, Kyambogo University, Kampala, Uganda; d Department of Information Systems, Makerere University College of Computing and Information Sciences, Kampala, Uganda; e Reproductive Health Program/Sexual and Reproductive Health research program, Population Council Kenya, Nairobi, Kenya; f Department of Population Medicine, Harvard Pilgrim Health Care Institute, Harvard T H Chan School of Public Health, Boston, Massachusetts, Suffolk County; g WHO AFRO Digital Finance Team, Organisation mondiale de la Sante pour Afrique, Brazzaville, Brazzaville, Congo; h Department of Health Policy and Management, University of Ibadan College of Medicine, Ibadan, Oyo, Nigeria; i Department of Epidemiology and Medical Statistics, University of Ibadan, Ibadan, Nigeria; j Department of Public Health, Felix Houphouet Boigny University, Abidjan, Côte d’Ivoire; k Universite Cheikh Anta Diop de Dakar Institut Universitaire de Peche et d’Aquaculture Dakar, Guediawaye, Senegal

**Keywords:** Digital payment, immunisation, health workers, barriers, enablers

## Abstract

**Background:**

Timely and consistent digital payment of health workers is crucial for improving the effectiveness of immunization campaigns and achieving the polio eradication goals by 2026. However, evidence on the enablers and barriers to digital payments in sub-Saharan Africa (SSA) is limited.

**Objective:**

To explore the enablers and barriers to digital payments for immunization campaign health workers in eight selected SSA countries.

**Methods:**

An exploratory case study using qualitative methods was conducted in eight SSA countries: four English-speaking (Uganda, Kenya, Nigeria, Ghana) and four French-speaking (Senegal, Ivory Coast, Cameroon, Democratic Republic of the Congo). A scoping review and in-depth interviews were conducted between March and May 2022 in each country. Data were analyzed thematically.

**Results:**

Digital payments are rapidly expanding in SSA, including during large-scale immunization campaigns. Key enablers included supportive regulatory frameworks, increasing mobile phone and digital platform coverage, and the benefits of digital payments. Barriers included inadequate telecom infrastructure, cybercrime, challenges with customer registration, higher transaction costs, and payment delays.

**Conclusion:**

This study identifies key enablers of digital payments in eight SSA countries, including supportive regulations, growing mobile phone ownership, and expanding digital platforms. Persistent challenges – such as limited infrastructure, verification constraints, and payment delays – affect implementation. Findings, while context-specific, offer valuable insights for policymakers to strengthen digital payment systems, improve verification, and enhance coordination to optimize health worker payments during immunization and public health campaigns.

## Paper Context

- **Main findings:** Digital payments are expanding rapidly in sub-Saharan Africa due to supportive regulatory frameworks and increased mobile penetration, but persistent telecom infrastructure gaps, registration challenges, and payment delays continue to affect timely remuneration of health workers during immunization campaigns.
- **Added knowledge:** This study advances understanding by specifically examining how digital payment systems intersect with the unique financial and operational realities of campaign-based health workers in large-scale immunization efforts in SSA.
- **Global health impact for policy and action:** Strengthening digital infrastructure, automating verification processes, and tailoring payment platforms to campaign contexts can reduce delays, improve health worker motivation, and enhance the effectiveness of immunization programs and pandemic preparedness strategies.

## Background

The success and effectiveness of immunization campaigns are largely influenced by the performance of health workers. However, the lack of appropriate incentives for frontline campaign workers remains a challenge for running successful campaigns [1]. Additionally, the motivation and subsequent performance of health workers are negatively affected by delayed and inconsistent payments [2]. Hence, initiatives that promote timely payments are crucial.

The Polio Eradication Strategy Framework 2022–2026 emphasises the importance of innovative strategies such as digital payments to enhance vaccination campaigns [3]. Digitized direct payments have been associated with timeliness and transparency in payments of health workers’ allowances and the effective implementation of immunization campaigns [4]. Recent experiences from COVID-19 cashless payments in West Africa driven by GAVI, Master Card, and Stump Trust showed that digitized payments improve health worker motivation and performance, potentially increasing vaccine coverage and related outcomes [5].

Large-scale immunization campaigns often involve the rapid deployment of large numbers of health workers – many of whom are temporary or contract staff hired specifically for the duration of the campaign. These workers are typically not integrated into regular payroll systems, and their operations span geographically dispersed and hard-to-reach areas [2,6]. These conditions make traditional cash payments logistically difficult, delay-prone, and vulnerable to fraud and leakage [7,8]. Digital payments – particularly via mobile money – provide a more efficient and transparent alternative, enabling timely compensation directly to health workers, regardless of their location [9]. Studies have shown that such timely, secure payments boost health worker morale, retention, and performance, which are vital to the success of these fast-paced campaigns [10].

In 2020, the WHO Afro region, supported by the Bill and Melinda Gates Foundation, through the Global Polio Eradication Initiative (GPEI) program, initiated a digital payments pilot program in 47 countries [11]. The aim was to improve the effectiveness of polio immunization campaigns and the timeliness of responses during the outbreak of vaccine-derived poliovirus. By 2021, this initiative had been adopted in many African countries, with others working to deploy mobile-based cash transfers in health programs other than polio [11]. Although digital payment technologies, such as mobile money platforms, are increasingly utilized in LMICs for health campaigns, adoption rates remain modest and vary across countries due to various challenges such as poor digital infrastructure and cyber-crimes among others [12,13].

It is reported that many African countries have fast growing mobile money systems, which presents an opportunity for the growth of digital payment to health workers. This has been supported by the enabling policy frameworks [14]. In Uganda, a shift has occurred from predominantly cash-based payment systems to digital payment platforms, mainly using MTN and Airtel Mobile Money, and banking agents which are supported by the government, with regulatory frameworks from the Bank of Uganda [15].

Similarly, Ghana’s payment system has undergone a series of systematic/strategic changes and has now progressively transitioned from paper based to e-payments. Nigeria has employed digital payment solutions like Paga and OPay to distribute salaries to health workers, with support from the Central Bank of Nigeria. In Senegal and Ivory Coast, with the high political support, digital wallets like Orange Money are increasingly used for disbursing payments in the healthcare sector. The Democratic Republic of Congo is seeing a rise in the use of mobile money, primarily through Vodacom’s M-Pesa, despite geographic challenges. Cameroon experienced similar trends with systems like MTN Mobile Money and Orange Money.

Kenya is reported to be at the forefront with M-Pesa, demonstrating the successful implementation of mobile money in healthcare with various pilots. Despite these advances, digital payment has been associated with several challenges. These include limited mobile network coverage, weak personal identification systems, cyber fraud, difficulties in purchasing equipment and software, difficulties in integrating digital payments into the ministries of health payment systems, and transaction costs [16].

Although digital payments to health workers have potential to improve performance of health systems and the effectiveness of campaigns as noted above, evidence regarding the enablers and barriers to digital payment across sub SSA remains scanty [17,18]. Therefore, this study aimed to document the enablers and barriers to digital payments in Africa. Understanding the enablers and barriers to digital payment of health workers in sub-Saharan Africa is crucial for optimizing the implementation of digital payment systems for health workers [13].

**Figure 1.**
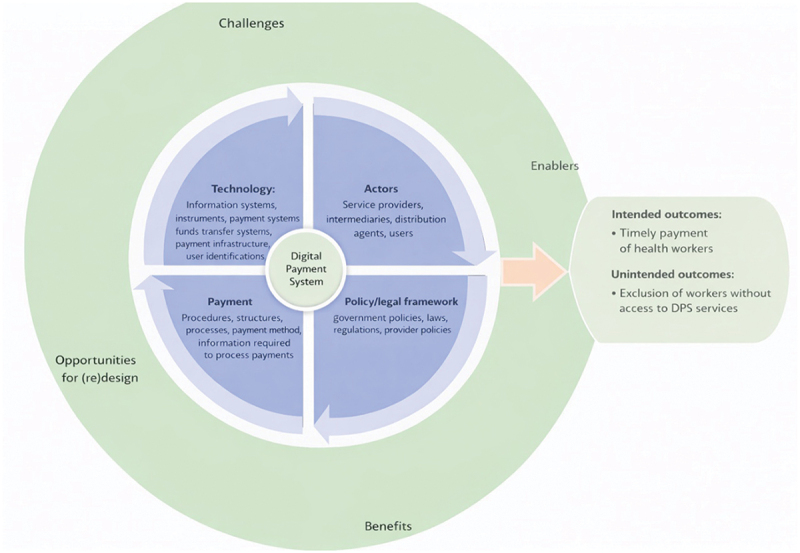
A conceptual framework of the digital payments ecosystem.

This landscape analysis was guided by theoretical frameworks for payment systems and digital payment ecosystems [19,20] which highlight the key features of the digital payments ecosystem namely 1) **Technology**: comprising information systems, payment instruments, funds transfer systems, payment ICT infrastructure, network, user identification systems, etc.; 2)**Actors**: intermediaries, service providers, distribution system/agents, system operators, and users (payer, payee, programs, organizations), i.e. those who prepare and authorize payments and those who maintain the data and support the network of computers; 3) Policy/legal framework: government policies, laws, and regulations, and provider policies. 4) **Payment**: procedures, processes, payment methods, information needed to process payments, and structures. The digital payments ecosystem presents benefits, challenges, barriers, and (re)design opportunities for payment systems, which informs the focus of this paper (Figure 1).

## Methods

### Study design:

This was an exploratory qualitative case study design that described the landscape of digital payment systems for health workers on immunisation campaigns in eight SSA countries; Uganda, Kenya, Nigeria, Ghana, Senegal, Ivory Coast, Cameroon, and the Democratic Republic of Congo (DRC). A scoping review was conducted for both peer reviewed and gray literature. The qualitative approach provided explanations and captured experiences related to the use of digital payments [21]. The case study approach was useful for understanding the local contextual issues that influence digital payment positively or negatively [22]. Data collection was sequential, beginning with the document review to explore the status and factors influencing digital payments, followed by the in-depth interviews which were conducted both virtually and face-to-face, depending on the local context. The data was triangulated for a comprehensive understanding of enablers and barriers to digital payment. Lastly, stakeholder workshops were held to validate the findings in each of the study countries.

### Data collection methods

#### Literature search and Review

In all countries, the study was preceded by a scoping review using both peer-reviewed articles and grey literature. Peer reviewed articles were searched from various online databases, including Google, Google Scholar, HINARI, WHO, Cochrane, PubMed, DOAJ, Embase, Medline, Web of Science, and Global Health web pages to identify (a) peer-reviewed papers (b) policy briefs, (c) reports, and (d) blogs on digital payment systems.

For PubMed, a search string with a combination of several key terms was used. These included:

(digital payment OR digital payments OR digital financ* OR digitised OR digitized OR epay OR epays OR e-pay OR e-pays OR ‘e voucher*’ OR ‘mobile money’ OR electronic payment OR electronic payments) AND (health work* OR ‘health personnel’[MeSH] OR ‘community health worker*’) AND (health program* OR immuni?ation program* OR vaccination program* OR immuni?ation campaign*) AND (Africa OR ‘Sub-Saharan Africa’)

**Figure 2.**
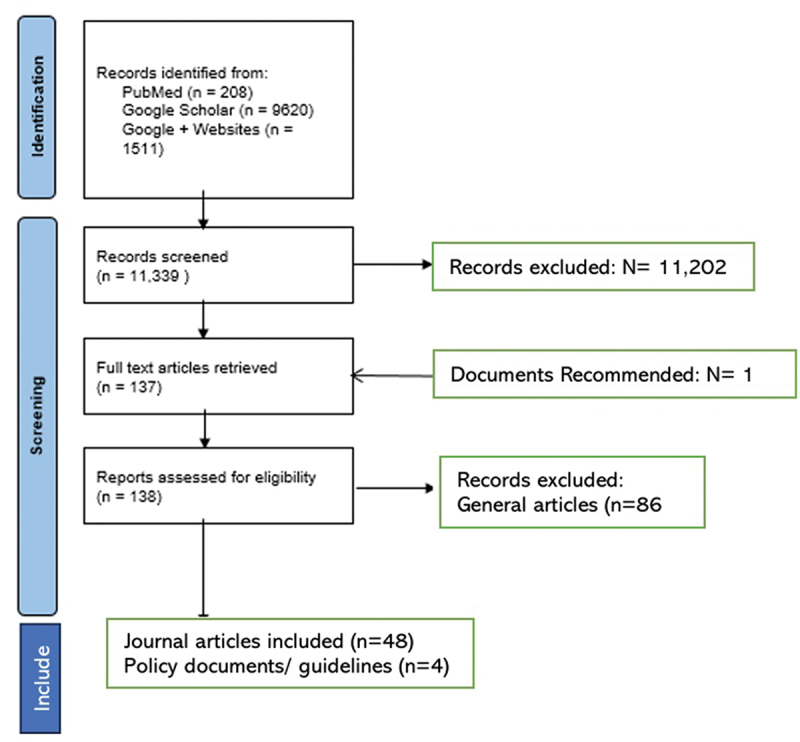
PRISMA flow diagram: description of identification of articles and documents.

For Google Scholar, the search terms included: 1. digital payments; 2. digitized payments; 3. electronic payments; 4. cashless payments; 5. health workers; 6. immunisation campaigns; 7. immunisation programs; 8. vaccination programs; and 10. vaccination campaigns. Only the first 100 hits were reviewed. The inclusion/exclusion criteria included all the literature available with full text in English or French on digital payments in SSA regardless of the year of publication. This is summarised in Figure 2. The screening and selection were based on the inclusion/exclusion criteria and ranked as high, moderate, and low priority. While we initially used a ‘high, moderate, and low priority’ ranking to guide selection, this was solely an internal categorization and does not represent standard scoping review terminology. High-priority literature aligned to the study questions, moderate-priority literature had broader application to the possible results that could be applied in the discussion, and low-priority literature was unrelated to the study and was therefore not considered.

Screened literature was reassigned to three reviewers for validation and re-ranking. The decision to include or exclude literature was made based on a concurrence between the first and second reviewers. In the event of differences in ranking, a third reviewer was assigned as a tiebreaker. Articles ranked high by two out of the three reviewers were included for a detailed review on the majority rule. Articles ranked either moderate or low by two of the three were excluded from the review by the majority rule.

### In-depth interviews

#### Participant selection and recruitment

In-depth interviews were conducted with key stakeholders involved in digital payment systems across the eight study countries (Uganda, Kenya, Nigeria, Ghana, Senegal, Ivory Coast, Cameroon, and the Democratic Republic of Congo). Stakeholders were purposively selected to capture diverse perspectives across the digital payment ecosystem, including health workers, policymakers/regulators, mobile money providers, financial institution representatives, implementing partners, and community-level actors.

A total of 130 participants were interviewed across all countries (Table 1). Within each country, potential participants were identified through professional networks, institutional affiliations, and stakeholder mapping. Individuals were contacted via email or telephone and invited to participate. All those approached agreed to participate; thus, no refusals were recorded.

**Table 1.** In-depth interview participants per country.

Country	Number of In-depth Interviews
Uganda	25
Kenya	10
Nigeria	17
Ghana	17
Senegal	21
Ivory Coast	14
Cameroon	22
DRC	18
Total	130

### Data collection procedures

Interviews were conducted between March and May 2022 by trained researchers in each country. Interviews were conducted in English or French, depending on the local context and participant preference. For non-English interviews, audio recordings were translated and transcribed into English by bilingual researchers. To ensure translation quality, transcripts were reviewed by a second bilingual team member, and discrepancies were resolved through discussion.

Interviews were conducted either face-to-face or virtually, lasted approximately 45–60 minutes, and followed a semi-structured interview guide developed based on the study’s conceptual framework. All interviews were audio-recorded with participant consent.

### Data management and analysis

All interview recordings were transcribed verbatim. A common codebook was developed collaboratively by the central research team and country leads, informed by the study objectives, conceptual framework, and emerging themes from initial transcript reviews. The codebook included both deductive codes (based on predefined themes such as enablers and barriers) and inductive codes (emerging from the data).

Qualitative data were analyzed using NVivo 12. A codebook was developed collaboratively by the research team to guide thematic coding. Interviews conducted in local languages were translated and checked for accuracy. Intercoder consistency was assessed by independently coding a subset of transcripts and resolving discrepancies through discussion. Cross-country synthesis was harmonized through iterative team discussions to identify common themes while retaining country-specific nuances.

### Cross-country synthesis

Following country-level analysis, coded data and thematic summaries were shared with the central analysis team based in Uganda. A central synthesis process was undertaken to compare and integrate findings across countries. This involved identifying common themes, as well as contextual similarities and differences in enablers and barriers to digital payments.

Findings from the interviews were triangulated with results from the scoping review and stakeholder validation workshops to enhance the robustness and credibility of the analysis.

## Results

### Scoping review findings

The scoping review was conducted following a structured and reproducible process. Literature searches were performed between March and May 2022 across multiple databases, including PubMed, Web of Science, Google Scholar, HINARI, Cochrane Library, Embase, Medline, DOAJ, WHO repositories, and relevant grey literature sources. Grey literature was identified through institutional websites (e.g. WHO, World Bank), policy reports, and targeted Google searches.

All retrieved records were exported into a reference management system, and duplicate records were identified and removed prior to screening. Screening was conducted in two stages: (1) title and abstract screening, and (2) full-text review. This process was performed independently by two reviewers, with disagreements resolved through discussion or consultation with a third reviewer.

Inclusion criteria comprised:

- Studies and reports focusing on digital payment systems in health programs
- Evidence related to health workers and/or immunisation campaigns
- Studies conducted in Sub-Saharan Africa
- Publications available in English or French

Exclusion criteria included:

- Studies not related to digital payments or health systems
- Studies outside Sub-Saharan Africa
- Articles without accessible full text

A total of 11,339 records were identified, of which 52 articles and policy documents met the inclusion criteria after full-text screening (see Figure 2: PRISMA flow diagram).

To support synthesis, included studies were systematically categorized based on relevance to the study objectives, rather than subjective ranking. Data from both peer-reviewed and grey literature were extracted using a standardized form capturing study characteristics (author, year, country, study type, key findings).

Findings from the scoping review were synthesized thematically and later integrated with qualitative findings from in-depth interviews and stakeholder workshops. The review informed the development of interview guides and provided a comparative evidence base to triangulate emerging themes.

Across the included studies, digital payment systems for health workers were reported to improve efficiency, transparency, and timeliness of payments. Mobile money emerged as the most widely used modality, supported by enabling regulatory environments and increasing mobile penetration. However, persistent challenges were identified, including limited infrastructure, identity verification barriers, transaction costs, and delays in disbursement, which were consistent across both literature and primary data sources.

### Digital payments for health workers

Findings are presented according to their source to enhance clarity. Evidence from the scoping review is reported first, highlighting trends and documented enablers and barriers to digital payments. Insights from in-depth interviews with health workers and key stakeholders are presented next, providing contextual and country-specific perspectives. Finally, themes validated and refined through stakeholder workshops are explicitly indicated, demonstrating triangulation and strengthening the credibility of the results.

Across the four English-speaking countries (Uganda, Kenya, Nigeria, Ghana), and four French-speaking countries (Senegal, Ivory Coast, Cameroon, and the Democratic Republic of the Congo (DRC)), digital payments are increasingly used in health-related programs to enhance efficiency, transparency, and timeliness in disbursing funds to health workers. Common programs leveraging digital payments include polio and routine immunizations, family planning, malaria control, HIV programs, drug administration, and national health insurance schemes. These digital systems have enabled funders, implementers, service providers, regulators, and beneficiaries to align the approaches that they use to improve service delivery.

Mobile money remains the most dominant digital payment modality, particularly in Uganda, Kenya, Ghana, and Nigeria. Bank transfers, digital wallets, and agent-based transactions also supplement payment systems, especially in urban areas. In Uganda, health workers engaged in ad-hoc campaigns were paid through mobile money using their registered National Identification Numbers (NIN). Kenya’s system relied heavily on mobile money for campaigns and involved a structured verification process before payment. Nigeria integrated digital systems such as IPPIS, ANRiN, and TARIBA to enroll both formal and ad-hoc health staff, linking payment with verification of tasks. Ghana emphasized digital payment in immunization programs but faced delays in disbursements.

Technologies used for payments include ATMs, point-of-sale devices, debit cards, mobile apps, and finance software. The adoption of digital payments has been driven by increased mobile phone ownership, the rise of mobile agents, improved policy environments, and digital interoperability among stakeholders. Enabling factors include policies such as Kenya’s E-money Regulation (2013), Uganda’s Mobile Money Guidelines (2013), and Nigeria’s regulatory framework for mobile services (2021). Digital payments offer several advantages: they are safer, faster, cost-effective, promote transparency, reduce corruption, and enhance health worker motivation and financial inclusion.

Despite these benefits, several challenges persist. Poor telecommunication infrastructure – especially in rural areas – hinders transaction reliability. Cybercrime and fraud raise concerns around security. Taxes on digital services, uneven distribution of agents, delays in problem resolution, and limited trust in third-party agents present significant barriers. Additionally, customer registration issues tied to ID documentation, the persistence of a strong cash culture (notably in Ghana), and delayed disbursements have affected adoption. French-speaking countries such as Senegal, Ivory Coast, Cameroon, and DRC face similar challenges but often contend with even more pronounced infrastructure and digital literacy gaps. Nonetheless, the region shows promising growth in mobile money uptake, with digital payments playing a crucial role in improving healthcare financing systems.

### Enablers of digital payment

The key enablers of digital payment were: conducive regulatory frameworks and government commitments, growing coverage of mobile phones and digital payment platforms and their benefits, as described below:

#### Conducive regulatory frameworks and government commitment

Across all countries, digital payments were supported by regulatory frameworks for digital payment and government commitment to promote and implement digital payments [12,23]. Some of the policies included; the Bank of Uganda Act, Kenya’s e-money Regulation 2013, the 2021 Regulatory framework for mobile money services in Nigeria, and the Ghana Digital Financial Services Policy, 2015. The interviews conducted across the study countries also indicated that the current digital regulatory framework governing the operation and management of digital payments was favourable for the implementation of digital payments for health workers in all countries, as shown in the quotes below:

*‘We have a very conducive digital payment regulatory environment with good policies and guidelines that have enabled digital payment to health workers to take off,’* **(Health worker, Uganda)**

*‘I see a lot of promise in the use of digital payment in every sector of the Ghanaian economy as the government has embraced digital payment through the adoption of relevant policies and walking the talk … ,’* **(Regulator/policy maker- Ghana)**

#### Increased ownership of mobile phones and network coverage

The growing ownership of mobile phones [12,23–27] and network coverage [27] at 94% ownership and 70% access to the internet across all the countries was a key enabler for the fast growth of digital payments. Data were drawn from GSMA Mobile Connectivity Index 2022 with denominators representing the adult population in each country. The reported values – 94% mobile phone ownership and 70% internet access – reflect aggregated estimates across the eight study countries, rather than precise figures for each country individually. Similarly, this was highlighted by the study participants from Uganda, where one noted:

*‘Everyone has a mobile phone at my workplace and I have not heard of anyone complaining about network issues. The mobile phone has made life easy because it is not only used for voice but a wallet in our pockets.’* **(Health worker, Uganda)**

#### Wide coverage of digital payment platforms

The study findings indicate a wide coverage with a growing rate of digital payment platforms across countries, albeit with limited access in some of the rural and hard to reach areas [11,23,27]. These include mobile money agents, banks, non-conventional financial institutions such as Microfinance and SACCOs, and aggregators. This has facilitated the growth of digital payments even in some remote locations. One of the respondents from Ghana noted:

*‘Wherever there’s coverage of at least one payment platform we’ve been able to extend digital financial services there.’ (* **Electronic Money Issuer- Ghana)**

#### The benefits associated with digital payments

Across all countries, the benefits of digital payments have motivated stakeholders to adopt digital payments [28]. These included: reduced operating costs reported in Ghana, Kenya, and Nigeria [11], fast and instant remittances in Senegal, DRC and Cote d’Ivoire [29], safety of the digital payment transactions [24], and convenience, flexibility, confidentiality, global focus on narrowing digital financial inclusion, and massive youth involvement [29].

#### Digital payment interoperability

In all the countries, digital payment systems allow customers to make transactions/receive payments across different digital payment systems, networks and providers within and beyond their country borders. This eases access to digital payments. For example, interoperability between the regulators, development partners/donors, mobile money service providers, and end users/implementers facilitated the uptake of digital payment for health workers in Africa [30].

#### Global concern for digital inclusion

The global focus on narrowing digital inclusion gaps between the banked and unbanked population was cited as prompting many countries to develop digital inclusion strategies favouring the adoption of digital payments by various countries including Ghana, Kenya, Nigeria, and Uganda [31,32].

### Barriers to digital payment

Several barriers were identified that affect the implementation of digital payments in SSA. These included: Poor digital payment infrastructure and coverage, cyber-crime concerns, transactional costs and delays, as elaborated below:

#### Poor digital payment infrastructure and coverage

Poor telecommunication infrastructure and coverage are reported as a major barrier to adoption of digital payments across the study countries. For instance, Nigeria and Cameroon are challenged by the weak digital financial inclusion and low mobile network coverage. Uganda is limited by the poor telecom infrastructure, especially in remote areas. The limited smartphone ownership in Kenya was highlighted as a key challenge, while DRC and Cameroon reported lack of adequate mobile phones especially among female beneficiaries and limited bank branches, which affect access to digital payments. Senegal and Ivory Coast struggle with inadequate payment platforms and poor network coverage especially in the remote areas, respectively. This is emphasized by some of the quotes below:

*‘The network coverage remains a key challenge … in all our sub-Saharan countries there are areas with no network.’* **(Ivory Coast Health worker)**

*‘Digital payment is good but, in our country, where we do not yet have a well-developed digital infrastructure, even at the level of health zones there is no connection, not everyone has a phone, this is a challenge and it delays payments.’* **(DRC Policy maker)**

#### Fraud and related challenges

Cyber fraud in digital payment transactions was highlighted to be a significant barrier to adoption of digital payments in Uganda, Kenya, Ghana, Ivory Coast, and Cameroon. Cyber fraud included cases where sensitive data falls into the wrong hands, leading to financial losses [19,33]. Key informants also highlighted concerns of fraud and fears that the funds may not reach intended recipients. For instance in Ghana, electronic money issuers noted that social engineering is the main factor for fraud in the industry. They explained that fraudsters capitalise on gullibility, negligence, naivety, acquiescence, and laxity on the part of their intended victims. One of the key informants from Ivory Coast noted:

*‘ … You must be discreet because there may be leaks. If you give your code to everyone, or someone has access to your code, you can be scammed too. This is still a challenge.’* **(Ivory Coast Vaccinator)**

*‘Someone called all of us on the same day when we received money and he claimed he was connected to this organisation, yet he was not! It surprised us and implied perhaps confidential information on the registration form was leaked.’ (***Uganda-Health worker**)

#### Extra operational and transactional costs

Fees and taxes, including those on personal and company incomes, licensing, and digital money transactions and withdrawal charges hinder digital payment uptake. Stakeholders from Uganda, DRC, Senegal, Ghana, and Kenya noted that the digital transaction fees and charges have steadily increased over the years. These fees included withdrawal charges and statutory taxes on digital money transaction and data bundles all of which diminished profit margins for mobile money agents, impacted the delivery cost of digital payment services and deterred adoption, particularly among the rural poor. Additionally, aggregators also charge high costs to transact funds on behalf of the implementers. Furthermore in Senegal, managers must follow up with beneficiaries after transfers have been made, adding an extra cost. However, organizations in Kenya and DRC reported covering most of the transaction costs. This was emphasized in the quotes below:

*‘ … Withdrawal fees were another thing that the beneficiary feared even when those fees weren’t exorbitant. However, when you tell someone that they are going to take $50, they would like to have all of it … For example, if you pay 50 dollars to a person, according to the VODACOM tariff, he has to pay 2 dollars to withdraw his money. When we transfer money to each other, we add $2.5 to make $52.5 and the person withdraws their $50.’* **(DRC Health Manager)**

*‘For certain activities, we can bear the costs but with enormous sums, we tell the person to go to the regions or if he agrees to bear the cost we will send the money.’* **(Senegal, Financier of the programme to fight AIDS)**

#### Customer registration process challenges

Some potential digital payment users face obstacles due to National Identification requirements, which are difficult to access in some African countries. This is further compounded by lack of unified databases and *KYC ‘Know your customer’* verification procedures to match the name of the worker registered in the database to the number provided [34]. Additional operational challenges in registering workers into the national database include lack of personal identity cards, name discrepancies, and difficulty obtaining SIM cards, particularly in DRC. Financial literacy gaps, especially regarding mobile lending apps also hinder uptake in Kenya and Uganda.

#### Delays in payments

In all countries, payment delays were noted as a gap that is affecting digital payments. The delays were associated with the manual verification processes, bureaucracies, system failures, and incorrect entries of personal data in the systems. For instance in Kenya and Uganda, delays occurred due to verification processes which were manual. In Ghana, it was noted that payments for health workers in EPI campaigns often delayed for weeks or even months mainly due to banking problems, incorrect employee details and bureaucratic processes. In Cameroon, shortcomings in the internal procedures of digital payment contributed to delays. This is emphasized in the quotes below:

*‘Some organizations experience significant delays in payments, lasting weeks or even months, causing communities to lose trust in organizers and digital payments … .This is attributed to the long bureaucratic procedures … ’ (***Kenya-Frontline manager**)

*“.Payments can range from a week to several weeks, or even a month … when payment is made to so many actors on the ground, 32,000 actors in the field where people have had trouble getting identified, payments delay …* **(Ivory Coast, Vaccinator)**

#### Limited trust in digital payments

Operational activities for digital payment systems are often managed by third-party agents, which can erode trust among potential users [24,25]. In Kenya, study participants reported that there is a section of stakeholders who do not trust digital payments; hence, they are less willing to adopt it. This was attributed to issues such as fraud, delayed payments, and sometimes missed payments. One of the key informants from Kenya noted:

*‘ … digital payment presents risks of payments going to the wrong recipients, sometimes lack of transparency; it is difficult to retrieve funds sent to the wrong person, which has made some beneficiaries lose trust.’ (***Kenya-Health manager**)

#### Limits in the transaction amount

One of the biggest constraints reported by both beneficiaries and managers and/or financial or accountants in Senegal is the capping of amounts that can be sent via the system. Mobile payment systems like (Orange Money and Wave) have ceilings that prevent the transfer or receipt of amounts above a certain threshold (200,000 FCFA for most platforms). Thus, managers were forced to look for solutions by changing accounts or distributing the money to different accounts. One of the key informants from Senegal noted:

*‘We’ve faced issues with capping Wave and Orange money accounts, limiting transactions to 200,000 FCFA. This causes problems when trying to send larger amounts. Sometimes, even as a sender, you may encounter limits, leading to failed transactions. To work around this, we’ve had to split payments between Orange money and Wave for the same recipient to bypass the ceiling.’* (**Senegal NTN Program Accountant**)

## Discussion

Unlike salaried staff within the health system, health workers recruited for immunization campaigns operate outside routine payroll structures, are often hired at short notice, and are deployed to remote locations. This makes cash-based compensation for these employees especially challenging and vulnerable to delays, fraud, and inefficiencies [2,8]. The implications are not just financial, but also programmatic. Delayed or missing payments contribute directly to absenteeism, reduced motivation, and compromised campaign performance.

This landscape analysis aimed to explore the enablers of and barriers to digital payment of health workers across sub-Saharan Africa (SSA), drawing lessons from eight representative English- and French-speaking African countries, including Uganda, Nigeria, Kenya, Ghana, Cameroon, Ivory Coast, the Democratic Republic of Congo (DRC), and Senegal. While the investigation of enablers and barriers may appear common to digital payments in general, this analysis is distinct in its application to temporary, mobile health workers engaged in campaign-based work [35,36].

Issues such as delays in payments due to manual verification, limited trust in third-party agents, or transaction limits impacting lump sum disbursements are more critical in short-term, high-turnover contexts like vaccination drives than in routine payroll scenarios [37]. In some settings, health workers expressed reluctance to trust third-party agents tasked with managing mobile money payments, particularly when these agents were unfamiliar or lacked visible accountability. This distrust was heightened when there was a lack of clear communication about payment timelines, deductions, or whom to contact in the event of an error [2,8]. For temporary workers unfamiliar with digital systems or operating in remote areas, third-party involvement without transparency contributed to fears of fraud due to previous experiences such as mobile money loans and payment errors [35]. Furthermore, the lack of robust identity verification and the digital exclusion of rural, often female, frontline workers presents a unique risk to health campaign effectiveness [36,38]. This highlights the need for targeted digital infrastructure investment and inclusive design of digital finance tools tailored to public health programs.

The study identified barriers such as inadequate telecommunication infrastructure limiting access to digital payment services in remote areas, cybercrime concerns, and payment delays [39]. These barriers are particularly detrimental in the context of large-scale immunization campaigns, where timely and complete compensation is directly linked to worker participation and campaign coverage [2,40]. Manual verification processes led to frequent delays due to incorrect records and a lack of integration with national ID systems. In several countries, health workers reported payment delays stretching for weeks, affecting morale and campaign success [8]. Transaction fees and mobile wallet limitations also posed a burden on health workers receiving modest stipends. In some countries, platforms had transaction caps that forced organizations to split payments across multiple providers, adding operational complexity and increasing the potential for errors [35,36].

Additionally, building trust in digital payment systems is crucial. Some health workers reported fears of fraud, often stemming from unfamiliarity with digital systems or prior negative experiences. Digital payment fraud has mainly been attributed to limited measures to curb cyber fraud as well as limited awareness among the beneficiaries [2,35]. Campaign-specific dynamics – short-term deployment, high numbers of new users, and limited training – exacerbate this distrust. Addressing cyber fraud risks and improving user education can support wider adoption.

The main enablers of digital payments included supportive regulatory frameworks, government commitments, and the growing coverage of mobile phone networks [36]. The existence of supportive regulatory frameworks for digital payments and government commitments were highlighted as major enablers for digital payment adoption consistently across all eight study countries and provided policy legitimacy and operational clarity, making it easier for implementing partners to coordinate digital disbursements at scale [39]. The increase in mobile phone ownership and expansion of digital payment platforms further enabled digital payment reach, even in rural regions. According to GSMA Intelligence [41], about 44% of the population in sub-Saharan Africa were mobile subscribers (unique subscriptions as a proportion of the population) by the end of 2023. This metric reflects subscription penetration rather than individual ownership or usage and is reported here to contextualize mobile access within the region.

These findings have implications for policymakers and implementers seeking to enhance the effectiveness of immunization campaigns by adopting digital payment. Enhancing digital infrastructure and automating verification processes can reduce payment delays. Ensuring digital payment platforms are tailored to the specific conditions of campaign-based health workers – such as low-income thresholds, intermittent payment cycles, and rapid onboarding needs – will help optimize performance [38,40]. While some of the issues highlighted, such as interoperability and digital inclusion, apply to other populations, their impact is heightened among health workers in campaign settings where any failure in the payment process can immediately affect service delivery. Hence, digital payment reform in immunization campaigns is not simply a technical solution but a cornerstone of effective health systems governance [2].

### The strengths and limitations

This study provides important insights into digital payment systems for health workers during immunization campaigns across eight sub-Saharan African countries. However, several limitations should be considered when interpreting the findings.

First, the purposive selection of countries may limit the generalisability of the findings beyond the study settings. While the selected countries provide representation across English- and French-speaking contexts, they may not fully capture the diversity of digital payment systems across all sub-Saharan African countries.

Second, the scoping review included both peer-reviewed and grey literature, and for Google Scholar, only the first 100 results were screened. This approach, while pragmatic, may have led to the omission of some relevant studies, particularly those not highly ranked by search algorithms.

Third, the screening process based on titles and abstracts may have resulted in the exclusion of potentially relevant studies where digital payments for health workers were not explicitly stated in these sections.

Finally, the data were collected in 2022, and given the rapidly evolving nature of digital payment systems in sub-Saharan Africa, some findings may not fully reflect more recent developments. Nonetheless, the study provides a valuable snapshot of the digital payment landscape during the study period and offers insights that remain relevant for similar contexts.

Despite these limitations, the study’s multi-country design and triangulation of data sources enhance the robustness and credibility of the findings.

## Conclusion

This study highlights key enabling factors for the adoption of digital payments across the eight sub-Saharan African countries included in this analysis, including supportive regulatory frameworks, increasing mobile phone ownership, and expanding digital payment platforms. These findings suggest that digital payment systems are gaining traction within the studied contexts, particularly in relation to large-scale immunization campaigns.

However, several persistent challenges remain, including inadequate telecommunications infrastructure, limited interoperability, registration and verification constraints, and delays in payments. These barriers continue to affect the efficiency and reliability of digital payment implementation for health workers.

Given the purposive selection of countries and the context-specific nature of the findings, the results of this study are most applicable to the settings examined and may not be generalizable to all sub-Saharan African countries. Nonetheless, they provide important insights for policymakers and implementers seeking to strengthen digital payment systems within similar contexts.

Future efforts should focus on strengthening digital infrastructure, improving verification systems, and enhancing coordination among stakeholders to optimise the effectiveness of digital payments for health workers in immunization and other public health campaigns.

### Author reflexivity Statement

As researchers conducting this landscape analysis on digital payments to health workers during large-scale immunization campaigns in sub-Saharan Africa, we acknowledge our positionality and potential influences on the study. Our team comprises individuals with diverse backgrounds in global health, digital finance, and health systems research, some with prior experience working with governments, donors, and implementing agencies in the region. While this expertise provided valuable context, we remained vigilant about mitigating biases by triangulating data from multiple sources, engaging diverse stakeholders, and ensuring rigorous methodological transparency. We recognize that our perspectives are shaped by our affiliations, academic training, and prior engagements with digital payment initiatives. To uphold objectivity, we prioritized the voices of frontline health workers and local decision-makers, ensuring their lived experiences informed our findings. Additionally, we critically reflected on power dynamics in data collection and analysis, striving to represent the realities of digital payment implementation accurately. Our goal is to contribute actionable insights that support equitable, efficient, and sustainable payment systems for health workers in sub-Saharan Africa.

## Data Availability

Data supporting the findings of this study are available from the corresponding author, VMK, upon reasonable request. Access may be granted for academic or research purposes, subject to approval by the study team and compliance with ethical and confidentiality requirements. Restrictions may apply to protect participant privacy and sensitive information.
